# Detection of *Streptococcus equi* subspecies *equi* using a triplex qPCR assay

**DOI:** 10.1016/j.tvjl.2012.07.007

**Published:** 2013-03

**Authors:** Katy Webb, Colin Barker, Tihana Harrison, Zoe Heather, Karen F. Steward, Carl Robinson, J. Richard Newton, Andrew S. Waller

**Affiliations:** Centre for Preventive Medicine, Animal Health Trust, Lanwades Park, Kentford, Newmarket, Suffolk CB8 7UU, UK

**Keywords:** Equine, Strangles, *Streptococcus equi*, Triplex quantitative PCR

## Abstract

Genome sequencing data for *Streptococcus equi* subspecies *equi* and *zooepidemicus* were used to develop a novel diagnostic triplex quantitative PCR (qPCR) assay targeting two genes specific to *S. equi* (*eqbE* and *SEQ2190*) and a unique 100 base pair control DNA sequence (*SZIC*) inserted into the *SZO07770* pseudogene of *S. zooepidemicus* strain H70. This triplex strangles qPCR assay can provide results within 2 h of sample receipt, has an overall sensitivity of 93.9% and specificity of 96.6% relative to the *eqbE* singlex assay and detects *S. equi* at levels below the threshold of the culture assay, even in the presence of contaminating bacteria.

## Introduction

Strangles, caused by *Streptococcus equi* subspecies *equi*, is the most frequently diagnosed infectious disease of horses worldwide and is responsible for significant welfare concerns and economic losses to the equine industry. The disease is characterised by abscessation of the lymph nodes of the head and neck. Abscesses formed in the retropharyngeal lymph nodes usually rupture into the guttural pouches, which drain via the nostrils, leading to the classical mucopurulent nasal discharge associated with strangles. Over time, the purulent material in the guttural pouches of some horses becomes inspissated, enabling *S. equi* to persist in horses that have recovered from the acute disease for up to several years in the absence of clinical signs (Newton et al., 1997).

*S. equi* is shed from persistently infected carrier horses periodically, allowing transmission to naïve individuals and resulting in new outbreaks of disease. The generation and persistence of carriers within equine populations is critical to the spread of *S. equi* infection. Efficient identification and treatment of carriers is important for prevention and eradication of this disease.

Diagnosis of *S. equi* infection has been based upon the culture of this β-haemolytic organism using selective media, followed by biochemical tests, which rely on the inability of *S. equi* to ferment trehalose, lactose or sorbitol (Bannister et al., 1985). Isolation of β-haemolytic colonies may be confounded by the presence of other bacteria, most notably *Streptococcus equi* subspecies *zooepidemicus* and *Streptococcus dysgalactiae* subspecies *equisimilis*, which may lead to the generation of false negative results. Isolation and identification of *S. equi* by routine methods is time consuming and requires a minimum of 48 h from receipt of clinical samples. This reporting delay often has consequences for the isolation of infected horses, providing *S. equi* with greater opportunity to transmit through naïve populations.

The first PCR-based test developed for *S. equi* targeted the 5′ region of the *SeM* gene. Using this target, up to three times more clinical samples were positive for *S. equi* than by culture and biochemical tests alone (Timoney and Artiushin, 1997; Newton et al., 2000). Historically, the *SeM* gene was thought to be non-variant, based upon its *Hin*dIII restriction pattern on Southern blotting (Galan and Timoney, 1988). However, it is now known that this region of the *SeM* gene is highly variable (Anzai et al., 2005; Kelly et al., 2006) and can be deleted in strains of *S. equi* isolated from persistently infected carriers (Chanter et al., 2000). The loss of diagnostic PCR targets leading to incorrect reporting can have deleterious consequences for the control of infectious disease. In Sweden, the occurrence of a 377 base pair (bp) deletion in CDS1 of the pSW2 plasmid of the human pathogen *Chlamydia trachomatis* resulted in the false negative diagnosis of many infected patients, and the rapid spread of this variant within the population (Seth-Smith et al., 2009).

Genes for the superantigens *seeH*, *seeI*, *seeL* and *seem*, encoded on the prophages φSeq4 and φSeq3, have potential as diagnostic candidates for *S. equi* subsp. *equi* (Baverud et al., 2007). However, the production of four superantigens confers a certain level of functional redundancy that could lead to loss of target sequences (Paillot et al., 2010). Furthermore, *S. zooepidemicus* multilocus sequence types ST-106, ST-118 and ST-120 were qPCR positive for *seeL* and *seeM* (Holden et al., 2009), suggesting that the use of these targets may lead to the identification of false positive results in some *S. zooepidemicus* strains. The gene encoding the factor H-binding protein Se18.9 has also been highlighted as a diagnostic target, but is present in ST-57 strains of *S. zooepidemicus* (Holden et al., 2009), suggesting that qPCRs targeting this sequence may also lead to false positive results.

To ensure the robust and sensitive identification of horses infected with *S. equi*, we have developed a triplex qPCR test that targets two *S. equi*-specific genes and employs an internal control strain of *S. zooepidemicus*, which serves as a DNA extraction and within-assay PCR control for every sample to reduce the risk of false negative reporting.

## Materials and methods

### Bacterial strains, media and growth conditions

*S. equi* strain 4047 (*Se*4047) was isolated from a submandibular lymph node abscess of a New Forest pony in 1990 and *S. zooepidemicus* strain H70 (*Sz*H70) was isolated from a Thoroughbred horse in 2000. Strains were cultured in Todd Hewett broth (Oxoid) at 37 °C with 5% CO_2_.

### Target selection

*S. equi*-specific targets were selected by use of comparative genome analysis of the published *Se*4047 and *Sz*H70 genomes (Heather et al., 2008; Holden et al., 2009). *eqbE* and *SEQ2190* were amplified by PCR using DNA samples previously extracted from 26 *S. equi* strains (Holden et al., 2009) and the primers ZM435/ZM436 and 2190A/2190B, respectively (Table 1). Products were sequenced on an ABI3100 DNA sequencer with BigDye fluorescent terminators using the same primers, with the addition of ZM437 for sequencing *eqbE*.

### Design and generation of the Sz07770c internal control strain

Two 500 bp fragments of the *SZO07770* pseudogene were amplified by PCR from *Sz*H70 using primers Ec07770Fwd1/Ec07770Rev1a and Ec07770Fwd2/Ec07770Rev2 (Table 1). PCR products were digested with *Eco*R1/*Pvu*1 and *Pvu*1/*Sal*1, respectively, and ligated into the multiple cloning site of the pGHost9 plasmid to generate pGHost9ΔSZO07770, which was sequenced using primers 5′pGhost9 and 3′pGhost9 (Table 1).

An artificial 82 bp DNA sequence with no significant homology with any nucleotide sequences on the NCBI database was designed (Table 1). Restriction sites for *Pvu*I and *Age*I were engineered to the 5′ and 3′ end of this DNA fragment, respectively, and the resultant DNA sequence, SZIC, was produced using the Gene Oracle gene synthesis service. The synthesised SZIC DNA was digested with *Pvu*I and *Age*I restriction enzymes and sub-cloned into pGHost9ΔSZO07770 digested with the same restriction enzymes, such that the resultant plasmid, pGHost9SZIC, contained *SZIC* flanked by 500 bp *SZO07770* gene fragments.

The pGHost9SZIC plasmid was transformed into electro-competent *Sz*H70. Integration of the construct, inserting the *SZIC* sequence into the chromosomal copy of the *SZO07770* gene and excision of the pGHost9 plasmid, was achieved by allelic replacement mutagenesis, as described for a Δ*prtM* mutant (Hamilton et al., 2006), creating the strain *Sz*07770c. The fidelity of *SZIC* was confirmed by PCR and sequencing across the *SZO07770* insertion site.

The *Sz*07770c strain was grown to an optical density of 0.3 at 600 nm in Todd Hewitt Broth and inactivated by heating at 95 °C for 30 min. No colonies of *Sz*07770c were obtained when 100 μL of this culture was incubated overnight at 37 °C in 5% CO_2_ on colistin-oxolinic acid blood agar (COBA) streptococcal selective agar (Oxoid). The killed culture was diluted to obtain a bacterial density equivalent to 80,000 colony-forming units (cfu)/mL in phosphate buffered saline and stored at −20 °C prior to use.

### DNA extraction for assay validation

One millilitre of excess clinical sample was spiked with 25 μL killed diluted *Sz*07770c, containing bacteria equivalent to 2000 cfu in the original live culture. DNA was extracted using the GenElute kit (Sigma) and eluted in 200 μL double distilled H_2_O.

### Development of the triplex qPCR assay

Compatible primers and minor groove binder (MGB) probes were designed against *eqbE*, *SEQ2190* and *SZIC* (Table 1). Reaction conditions were optimised for a fast cycling assay using an ABI StepOne Plus instrument using KAPA Fast probe mix (Kapa Biosystems) in a ratio of 50:50 master mix:DNA sample. Each reaction contained 10 μL KAPA Fast probe mix, 450 nM *eqbE*2 forward, 450 nM *eqbE*2 reverse, 450 nM 2190 forward, 450 nM 2190 reverse, 200 nM *SZIC* forward, 200 nM *SZIC* reverse, 150 nM *eqbE2* probe, 2190 probe and *SZIC* probe and 10 μL DNA extracted from the clinical sample. Thermal cycling conditions were 3 min at 95 °C, followed by 40 cycles of 95 °C for 3 s and 60 °C for 10 s. All qPCR experiments were performed in triplicate.

### Validation of the triplex qPCR assay and comparison with the eqbE singlex qPCR assay

Clinical samples (*n *= 213; >2 mL) received by the Animal Health Trust (AHT) diagnostic laboratories for detection of *S. equi* via the *eqbE* singlex qPCR assay were used in this study (see Appendix A: Supplementary Table 1). Two 1 mL aliquots of the original clinical sample were removed and blinded. Heat-killed *Sz*07770c (25 μL) was added to one aliquot and both aliquots were then centrifuged at 16,100 *g*. DNA was isolated from the pellets using the GenElute kit (Sigma). Samples containing spiked *Sz*07770c were analysed using the triplex qPCR assay and the number of copies of *eqbE* in the DNA isolated from the other aliquot was quantified by qPCR using the *eqbE* singlex assay by the AHT diagnostic laboratory.

### *Streptococcus equi* culture test

Clinical samples (*n* = 194) submitted to the AHT diagnostic laboratory were tested for *S. equi* by routine culture and identification.

### Statistical analysis

Receiver operator characteristic (ROC) curves, sensitivity and specificity of the qPCR and culture assays were calculated using STATA (StataCorp LP). Two-tailed Spearman’s Coefficients were calculated using PASW (version 18, SPSS). A two-tailed unpaired student’s *t* test was used to determine the significance of continuous data.

## Results

### Conservation of eqbE and SEQ2190 targets

PCR and sequencing of a 1063 bp region of *eqbE* (positions 1,229,518 to 1,228,456 of the *Se*4047 genome) and a 449 bp region of SEQ2190 (positions 2,203,349 to 2,203,797 of the *Se*4047 genome) demonstrated that these sequences were identical across all 26 strains of *S. equi* examined.

### Generation of the Sz07770c strain

PCR and sequencing across the insertion site confirmed that the *SZIC* sequence had been incorporated into the *SZO07770* pseudogene in the *Sz*07770c strain and that all plasmid DNA had been excised.

### Limit of detection of the triplex qPCR assay

**Fig. 1:**
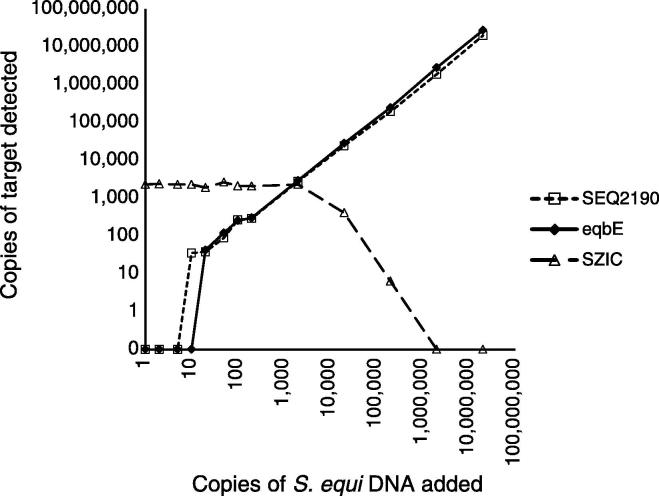
Effect of increasing concentration of *S. equi* DNA in the triplex qPCR reaction**.** Dilution series of *Se*4047 genomic DNA in the presence of 2000 copies of *SZIC* internal control target.

Quantification of *eqbE*, *SEQ2190* and *SZIC* in samples containing a dilution series of *Se*4047 DNA and approximately 2000 copies of DNA from *Sz*07770c determined that the triplex qPCR assays had a limit of detection of 20 copies (*eqbE* assay) and 10 copies (SEQ2190 assay) of *S. equi* DNA, respectively (Fig. 1). Increasing the quantities of *Se*4047 DNA above 10,000 copies led to competition and inhibition of the SZIC assay (Fig. 1).

### Validation of the triplex qPCR assay

**Fig. 2:**
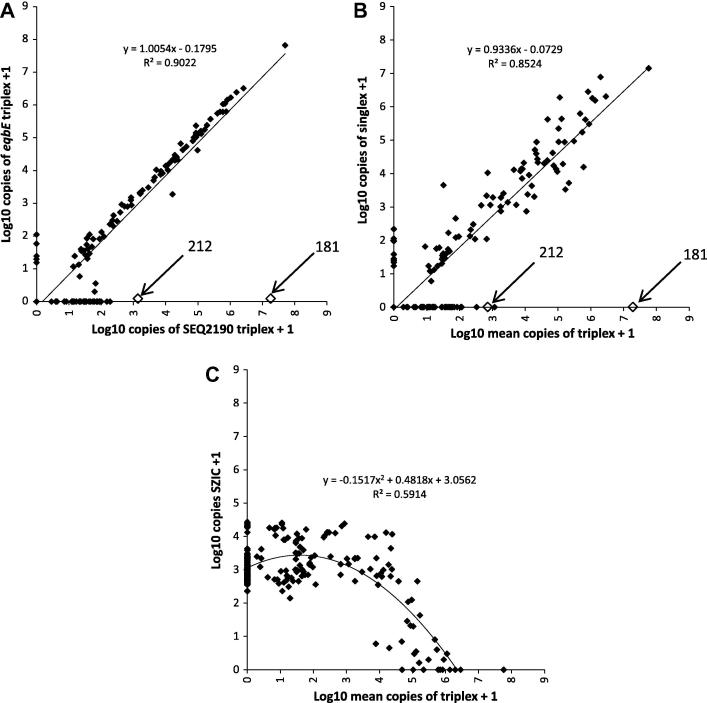
(A) Relationship of log_10_ qPCR copy numbers for *eqbE* and *SEQ2190* within the triplex assay. The location of the results for samples 181 and 212 are highlighted. (B) Log_10_ mean quantity of *S. equi* DNA detected in the triplex assay compared with *eqbE* quantified by the singlex assay. The location of the results for samples 181 and 212 are highlighted. (C) Effect of increasing quantities of *S. equi* in clinical samples on log_10_*SZIC* copy number. A copy number of one was added to all data, which was then transformed into a logarithmic format for presentation purposes. The line of best fit and its equation is shown on each graph.

Sixty-six of 213 clinical samples tested positive for *S. equi* via the *eqbE* singlex qPCR assay (*eqbE* copy number ⩾100) and 147 tested negative. Blinded details of all clinical samples tested, including culture, singlex and triplex assay results, are presented in Supplementary Table 1 (see Appendix A). Copy numbers for *eqbE* and *SEQ2190* quantified within the triplex assay correlated well with each other (Fig. 2A) and generated a Spearman’s Coefficient of 0.84 (*P* < 0.001). Mean triplex assay copy numbers also correlated well with the singlex *eqbE* results (Spearman’s Coefficient of 0.80, *P* < 0.001) (Fig. 2B).

Sample 181, recovered from the guttural pouches of a horse in Dorset, contained *SEQ2190* copy numbers of 14,030,667, but did not amplify *eqbE* in the triplex or singlex assays. Sample 212, taken from the guttural pouches of another horse from the same yard in Dorset, similarly tested negative for *eqbE* using both assays, but was positive for *SEQ2190* (Fig. 2A and B). These samples were removed from the data set for the purposes of plotting the associated lines of best fit.

Five clinical samples (2.3%) originally tested negative for the *SZIC* control, highlighting a potential failure in the DNA extraction method. These samples were re-extracted, retested and the *eqbE* and *SEQ2190* triplex results for two discrepant samples, 194 and 211, then were found to be in agreement with singlex assay results.

The *SZIC* assay detected a mean of 3994 copies in all reactions where the amount of *S. equi* DNA was ⩽10,000 copies (*n* = 175) (Fig. 2C). However, the *SEQ2190* and *eqbE* assays significantly interfered with the *SZIC* assay when the amount of *S. equi* DNA exceeded 10,000 copies per reaction (*P *= 0.0038), leading to a mean of 940 copies of SZIC in these clinical samples (*n *= 38) and a non-linear line of best fit (Fig. 2C).

To identify the optimal assay breakpoints and so ensure that the most robust diagnosis was obtained, triplex data were compared with the singlex qPCR assay as the gold-standard using STATA. Optimal sensitivity (90.9%) and specificity (96.6%) for the *eqbE* component of the triplex assay were achieved with a breakpoint of 110 copies, generating an area under the ROC curve of 0.95, where a value of 1.0 indicates complete concordance of data. Optimal sensitivity for the *SEQ2190* component of the triplex assay (sensitivity 92.4%, specificity 97.3%) was achieved with a breakpoint of 150 copies, generating an area under the ROC curve of 0.9727.

Using these breakpoints and combining the results of the *eqbE* and SEQ2190 assays, so that a sample was reported as positive if either assay reached its cut-off, 67 positive samples were identified, of which five were negative by the singlex assay, giving an overall sensitivity and specificity for the triplex assay of 93.9% and 96.6%, respectively.

### Comparison of qPCR with the culture assay

The culture test identified no additional positive clinical samples and had an overall sensitivity of 60.3% and specificity of 100% relative to the combined qPCR results. Other β-haemolytic streptococci were present in 15/27 (56%) culture negative, qPCR positive samples (10 contained *S. zooepidemicus* and five contained *S. equisimilis*; see Appendix A: Supplementary Table 1). The mean qPCR copy number of culture positive/qPCR positive samples was 1,388,580 (*n* = 41). Culture negative, qPCR positive samples that did not contain other β-haemolytic streptococci had a mean copy number of 415 (*n *= 12) and culture negative/qPCR samples that contained other β-haemolytic streptococci had a mean *S. equi* DNA copy number of 170,312 (*n* = 15).

## Discussion

Emerging genome sequencing data and an extensive collection of *S. equi* and *S. zooepidemicus* isolates were exploited to select two conserved *S. equi*-specific targets that are located in separate regions of the *Se*4047 genome. The *eqbE* gene forms part of the equibactin locus, which encodes a non-ribosomal peptide synthesis system that enhances the ability of *S. equi* to acquire iron (Heather et al., 2008). *SEQ2190* encodes a putative sortase-processed protein, which was conserved in all strains of *S. equi* examined. Targeting both *eqbE* and *SEQ2190* is predicted to significantly reduce the likelihood that both diagnostic targets are lost in a strain, resulting in the failure of the test and subsequent clonal expansion of the modified strain.

This risk and the usefulness of the triplex assay are highlighted in samples 181 and 212, which tested negative for *eqbE*, but positive for the *SEQ2190* gene. Decay of the *SeM* gene in isolates recovered from the guttural pouch has been reported previously (Chanter et al., 2000). Recently, we identified significant decay of the *S. equi* genome on sequencing >230 isolates using the Illumina HiSeq platform (data not shown), which suggests that reliance on only one diagnostic target is likely to lead to the reporting of false negative results.

The novel internal control strain, *Sz*07770c, which serves as a DNA extraction and assay control, ensures that all clinical samples should generate a qPCR result. High levels of *S. equi* DNA (>10,000 copies) out-compete the *SZIC* assay and generate *eqbE* positive/*SEQ2190* positive/*SZIC* negative results. Similarly, high levels of *Bacillus anthracis*, *Francisella tularensis* and *Yersinia pestis* inhibited the detection of a *Bacillus thuringensis* internal control in a multiplex qPCR assay (Janse et al., 2010). The triplex qPCR assay was optimised to ensure that amplification of low concentrations of *S. equi* DNA (<1000 copies) was not inhibited by the presence of *SZIC*. The close agreement between the singlex and triplex assays in this study (95.8%) and low limit of detection (10–20 copies of *S. equi* DNA) suggests that no compromise on sensitivity was made through inclusion of the SZIC control.

The inclusion of the SZIC assay permits robust quality control of all assay stages for each clinical sample. Five clinical samples analysed using the triplex assay failed these criteria and were re-tested, two of which were positive. The five discrepant clinical samples that tested negative on singlex, but positive on triplex, similarly may be due to assay failures that would have gone unnoticed using the singlex assay system. Therefore, the triplex assay will reduce the number of false negative results and so enable the detection, isolation and treatment of infected horses before they can transmit *S. equi* to others. Since the triplex assay requires only the addition of primers and probes specific to SEQ2190 and SZIC, the extra cost per sample is £0.16,^2^ which enables the implementation of the triplex assay without additional cost to clients.

Comparison of results from the singlex and triplex qPCR assays with the culture assay highlighted a lack of sensitivity of the culture assay (60.3%). The lowest mean quantity of *S. equi* DNA in a culture positive sample was 960 copies. The presence of other β-haemolytic streptococci accounted for more than half (15/27) of culture negative, qPCR positive samples, emphasising the problem of isolating *S. equi* from mixed cultures. Our data demonstrate that the *S. equi* culture test can no longer be considered the to be gold-standard test for this organism.

## Conclusions

The use of sensitive qPCR assays will improve the identification and treatment of horses infected with *S. equi*, particularly carriers where intermittent low level shedding of this bacterium is common and can be missed by traditional culture and PCR assays. The strangles triplex assay provides a rapid, sensitive and robust method for the detection of *S. equi* infection for minimal additional cost.

## Conflict of interest statement

The use of *eqbE*, *SEQ2190* and the *SZIC* control for the detection of *S. equi* has been patented (US 2011/0201007, US 2007/0243195 and GB1122121.5).

## Figures and Tables

**Table 1:** Primer and probe sequences.

Primer/probe name	Sequence (5′–3′)^a^	Label/restriction site
ZM435	CCGAATTTGTCCAAGTGGTATG	
ZM436	GCACTCCGTTATACTCACTG	
ZM437	TTTGCTAGTGCTACTCCTGC	
2190A	ATGGGAACAGGACTACTTG	
2190B	GTCTTAGCTTCCTCTTTCGC	
Ec07770Fwd1	GACGACGAATTCTGAGAGGCAAGTGACGAGTC	*Eco*RI
Ec07770Rev1a	GACGACCGATCGGACGACACCGGTTTGCCAAACTCCCTTCCAAG	*Pvu*I, *Age*I
Ec07770Fwd2	GACGACCGATCGCACTTGCTTGTTCTAGCTGAG	*Pvu*I
Ec07770Rev2	GACGACGTCGACGGAACGAACCTCTTACCACA	*Sal*I
5′pGhost9	TTGGAAAGTTACACGTTACTAAAG	
3′pGhost9	GGGCGAATTGGGGTACCGGGC	
*eqbE*2 forward	TGGGATTCTGTGCCGATTTT	
*eqbE*2 reverse	CCCTGAAAGCATCACAATTCTAAA	
2190 forward	CAACGCGTAGAAGAACGATCTAAA	
2190 reverse	CCTCCAATTGAGCTTTTTGGTT	
SZIC forward	CGCATGCGGGTAGATTATGTAG	
SZIC reverse	TCCCACGAGAAGGTCGAGAA	
*eqbE*2 probe	ATTGTTACTATGGCTGAAGGT	FAM
2190 probe	AAGCCAAGGAAGCCACT	VIC
SZIC probe	AGAGACATCCAGGTCAA	NED
Artificial 82 bp DNA sequence	CCGTGTATTACGCATGCGGGTAGATTATGTAGGTAGAGACATCCAGGTCAAGTTCTCGACCTTCTCGTGGGAGGTGAACCAG	

a Restriction sites are underlined.

## Footnotes

2 UK£1.00 = US$1.55 = €1.24 (13 June 2012).
